# Knowledge, attitude, and behaviours on diet, physical activity, and tobacco use among school students: A cross-sectional study in two Indian states

**DOI:** 10.12688/f1000research.51136.1

**Published:** 2021-07-07

**Authors:** Shalini Bassi, Deepika Bahl, Melissa Blythe Harrell, Neha Jain, Arun Kandasamy, Subhash R. Salunke, Vinod Gajanan Shah, Prema Raghunathan, Selvarajan Markandan, Pratima Murthy, Monika Arora

**Affiliations:** 1 Health Promotion Division, Public Health Foundation of India (PHFI), Gurgaon, Haryana, 122002, India; 2 School of Public Health, Michael & Susan Dell Center for Healthy Living, The University of Texas Health Science Center at Houston, Austin, Texas, 78701, USA; 3 Department of Psychiatry, National Institute of Mental Health And Neuro Sciences (NIMHANS), Bengaluru, Karnataka, 560029, India; 4 Indian Institute of Public Health, Bhubaneswar, Odisha, 751013, India; 5 Janaseva Foundation, Pune, Maharashtra, 411030, India; 6 Department of Paediatrics, Rajarajeswari Medical, College & Hospital, Bengaluru, Karnataka, 560074, India; 7 Department of Health and Family Welfare, Government of Karnataka, Bengaluru, Karnataka, 560008, India

**Keywords:** Non-communicable diseases, risk factors, school, children, lifestyle related behaviours, health promotion

## Abstract

**Background:** Non-communicable diseases (NCDs) are escalating in India and can be attributed to behavioural risk factors such as unhealthy diet, physical inactivity and tobacco use that began in early years. Understanding adolescents’ knowledge, attitudes and behaviours (KAB) related to NCD risk factors would inform the development of school-based health programmes to prevent NCDs.

**Methods:** Sixth-grade students (n=1026) in 20 schools (10 private, 10 public) from two Indian cities (n=667 from Pune; n=359 from Bengaluru) participated in a KAB survey in 2019. Differences in KAB by gender, school type within cities were investigated.

**Results:** Knowledge about the harms of tobacco use was higher than knowledge about a healthy diet and the importance of physical activity. Only a small proportion of students did not eat breakfast (8.7%) or fruits (11.3%) daily. Only 33.4% of students read nutrition labels before choosing their food. Moderate-to-vigorous physical activity of less than an hour per day was reported by 42.5% of students. Approximately one-third of students had ever tried smoking tobacco (30.1%), smokeless tobacco (30.5%), and e-cigarettes (32.4%). Differences in these behaviours by gender and school type showed that both boys, girls and students of private and public schools are vulnerable.

**Conclusions:** The findings highlight that knowledge is low for thematic areas like diet and physical activity. Low knowledge can be attributed to unfavourable behaviours like lack of reading nutrition labels and indulgence in sedentary activities. To protect India’s young population (adolescents), there is a need to amplify health education activities and context-specific health intervention materials for them by engaging parents and communities. Thus, these programmes should be incorporated into the curriculum as part of the regular teaching, as they may induce positive changes in their knowledge and behaviours. In India, school health programmes should dedicate significant time to health promotion and NCD risk prevention.

## Introduction

The rising epidemic of non-communicable diseases (NCDs), such as cardiovascular diseases, cancers, diabetes and chronic respiratory diseases, is recognised as a leading cause of mortality and morbidity across all age groups in India and globally. ^1^ NCDs are responsible for more deaths in India each year than all other causes of death combined. ^2^ In the year 2019, 9.3 million deaths in India were attributed to all cause mortality, of which 6.1 million (64.9%) were due to NCDs. ^3^

Most NCDs are attributable to potentially modifiable behavioural risk factors, including unhealthy diets, physical inactivity, tobacco and alcohol use. ^4^ Synergistic effects of demographic transition, globalisation, and economic growth have resulted in an environment where more children and adolescents are exposed to and/or engage in these behaviours than ever before. ^5^ It is a well-known fact that lifestyle-related behaviours are often formed early and persist throughout life. ^6, 7^ Evidence has shown that 70% of premature deaths in adults due to NCDs are associated with risky behaviours that began in childhood. ^8^ Primordial prevention, including community-based intervention to promote healthier lifestyles, is extremely important for national development and to achieve the Sustainable Development Goals by 2030. Investment at an early age can yield a triple dividend of benefits, i.e., improving health later during adolescence, enhancing health throughout the course of life, and eventually contributing to the health of the next generation of children. ^9^ By 2030, India is expected to be the first country in the world to become home to more than 1.5 billion people, and its population is on target to reach 1.7 billion by 2050. ^10^ Thus, protecting and supporting child and adolescent health in this country is paramount not only to India’s overall well-being, but also to global health and economy.

Schools have been widely recognised as an important location for promoting healthy behaviours among children and adolescents. ^11, 12^ Incorporating health-based programmes into the school curriculum can substantially influence both health promoting behaviours and educational achievements. The above outcomes can be achieved by providing children and adolescents with adequate knowledge and skills, allowing them to establish healthy attitudes and social norms through such a curriculum. ^13^ Understanding their current knowledge, attitudes and lifestyle-related behaviours would immensely help both the health and education sectors in developing and implementing effective and efficient school-based health lifestyle programmes.

We have developed Project PaTHWay, a three-year, school-based programme (2018-2021), to promote healthier lifestyle practices among children. ^14, 15^ The overall objective of the programme is to prevent and control the risk factors of NCDs by delivering a school-based health lifestyle programmes to students from two cities in India. Specifically, the programme aims to addresses key behavioural NCD risk factors, such as unhealthy diet, physical inactivity, and tobacco use among school children. The students followed for three years in the project PaTHWay. The first year of the programme included, baseline knowledge, attitude, and behaviour (KAB) assessment and development of the health education programme based on the KAB assessment. Subsequently, school-based health lifestyle programme is being implemented for two years, followed by endline evaluation. In this paper, we present the baseline KAB of students relevant to these NCD behavioural risk factors, which have helped us develop Project PaTHWay school intervention.

## Methods

### Study design

A cross-sectional study was conducted during January- March 2019 in schools of Pune and Bengaluru to understand the KAB of school students related to diet, physical activity and tobacco use.

### Setting

The programme is being carried out in two cities of India, namely Pune (Maharashtra) and Bengaluru (Karnataka). Pune is situated in Western India, while Bengaluru is situated in Southern India. The number of NCD related deaths in both Maharashtra (70.6%) and Karnataka (72.4%) is higher than the national average of all Indian states (64.9%), across all age groups and both sexes. ^16^ Both Pune and Bengaluru are densely populated and highly urbanised cities. The total population of Pune is 9,429,408, of which there are 4,924,105 males and 4,505,303 females. The average literacy rate is 86.2%. ^17^ Similarly, Bengaluru is home to 9,621,551 individuals, of which there are 5,022,661 males and 4,598,890 females, and the average literacy rate is 87.6%. ^18^

Twenty schools (n = 10 public and n = 10 private) in Pune and Bengaluru were selected to implement Project PaTHWay. These schools were purposely selected to represent different socioeconomic strata (private schools: middle to higher socioeconomic status; public schools: lower socioeconomic status) within the two cities. The selected schools had common characteristics, such as each school having a division of the sixth grade into not more than one to two sub-sections, availability of playgrounds and equipment for physical activity, and provision of a school meal programme (public schools only).

### Participants

Students from these 20 schools, enrolled in the sixth grade (n = 1238; Pune: n = 806; Bengaluru: n = 432) were eligible and invited to participate in the baseline KAB questionnaire administered in 2019. Total sample of 1238 students (Pune: n = 806; Bengaluru: n = 432) were included in the present study.

### Instrument and measures

A self-administered, baseline questionnaire was implemented, to assess the students’ KAB related to NCD behavioural risk factors (unhealthy diet, physical inactivity, and tobacco use). The questionnaire included a section on socio-demographic profile of the student on aspects such as age, gender as well as education and occupation of their parents and other section on KAB. The diet related knowledge was measured with six questions, each related to elements of a balanced diet (e.g., *How many times per week should one eat breakfast?*). Knowledge about physical activity was measured with five questions (e.g., *How many minutes of physical activity should people of your age have daily?*); and tobacco use knowledge was assessed with 16 questions (e.g., *Does using chewing tobacco cause oral cancer?*).

Attitudes towards diet (e.g., *It is important for me to eat breakfast daily*), physical activity (e.g., *Taking part in physical activities can help me get better marks at school*) and tobacco use (e.g., *Using tobacco makes a person appear to be braver and more grown-up*) were measured using a 5-point Likert scale - strongly agree, agree, not sure, disagree and strongly disagree. Cronbach alpha was calculated for this construct as the mean score was calculated and presented for attitude towards diet, physical activity, and tobacco use. A higher diet and physical activity score denotes a positive attitude towards a healthy diet and physical activity, indicating lower risk of indulging in risky behaviours. A higher score for tobacco indicates that the adolescents have a less favourable attitude towards tobacco use, indicating a lower risk of tobacco consumption.

The questionnaire also included the behaviours specific to diet (e.g. *In a week, on how many days do you eat breakfast?*), physical activity (e.g., *How many hours do you spend doing moderate to vigorous physical activities?*), and tobacco use (e.g., *Have you ever tried a cigarette/beedi/hookah - even once or twice?*). Susceptibility to tobacco use, for both smoking tobacco and smokeless tobacco was also assessed using eight items (e.g. *Do you think you will smoke any type of tobacco when you enter college*?).

This questionnaire was developed based on the socio-ecological model ^19^ and adapting measures from reliable instruments that have been validated with adolescents in India. Surveys that we referred were, Global Youth Tobacco Survey (GYTS-2010), ^20^ Global Adult Tobacco Survey (GATS-2016-17), ^21^ Project EAT, ^22^ SPAN survey ^23^ and surveys used by the author of this study in previous studies conducted in India. ^24– 26^ An English version of the questionnaire was administered in private schools, and Kannada and Marathi versions were administered in public schools in Bengaluru and Pune, respectively. The questionnaire was pre-tested to assess its validity (face and content) and reliability (internal reliability of attitude construct).

For content validity, feedback was obtained from experts (n = 5) from multi-disciplinary field. These national and international experts have more than two decades of experience in psychology, epidemiology, public health and nutrition. Based on their feedback, the sequence of the questions was modified, more relevant questions were incorporated and language edits were made to simplify the questionnaire for students for better comprehension. To assess face validity, focus group discussions were conducted with students (n = 80) of grade six from both cities. These 80 students were selected from two schools in each city (one public and one private). To assess reliability, the questionnaire was administered with another 177 students from both cities. These schools or the students who were involved in face validity and internal reliability were not part of the main study. Based on students' feedback and internal reliability findings, language of the questionnaire was simplified, repeated and offending questions were deleted, options for questions were changed and added, detailed marking instructions were added to the questionnaire. Revised questionnaire was administered by a trained research team of the project. The questionnaire was administered during school hours, using a standardised protocol. Students were given unique identification codes to ensure confidentiality. Copies of the questionnaire in all three languages (English, Marathi, and Kannada) are available in *Extended data*. ^65^

### Ethics statement

Permissions for implementation of the study were obtained from the Maharashtra State Board of Secondary and Higher Education and Karnataka Secondary Education Examination Board. We also received permission from authorities at schools, written active informed parental consent, and student assent, indicated by a signature on the consent form. Information sheets were sent from the schools to the parents of all eligible students, wherein details of the study and questionnaire were outlined. The consent stated the permission for data collection, scientific publications, dissemination in conferences by maintaining the confidentiality of the study participants and anonymity of the collected data. Ethical approval for this study was obtained from the Institutional Ethics Committee of both PHFI (TRC-IEC-373/18) and NIMHANS [NIMHANS/EC (BEH.SC.DIV.)].

### Statistical analysis

Statistical analyses were performed using STATA Version.13.1 software. ^27^ The descriptive data were expressed as mean with standard deviation (SD) or proportions (%). A chi-square test was performed to examine differences in the categorical variables by socio-demographic factors (gender, school type). The summary scores for all attitude scales by gender and school type within each city were compared using the *t*-test. All statistics were analysed through a two-sided test; p-value less than or equal to 0.05 was considered statistically significant. Participants with missing information, parent refusal, student refusal or absenteeism on the day of questionnaire administration were excluded.

## Results

82.8% of the recruited sample participated in the baseline questionnaire (n = 1026 out of 1238). The reasons for non-participation included absenteeism (n = 112; boys: 67, girls: 45) and parent refusal (n = 100, boys: 54, girls: 46). The final sample size was 1026 (Private: 518 and Public: 508) consisting of 61.1% boys and 55.1% of the sample was from private schools. The mean age of the students recruited for the study was 12.5 ± 0.75 years. The full, de-identified dataset of student responses is available in *Underlying data*. ^64^

### Knowledge

*Diet*

Overall, students’ knowledge about healthy dietary practices was low ( Table 1). Many students (67.5%) knew that breakfast was the most important meal of the day. Only a few students (6.8%) knew they should eat at least five servings of fruits and/or vegetables a day. Knowledge about healthy dietary practices was generally highest among girls and private school students. In Pune, more girls (68%) knew breakfast was the most important meal of the day compared to boys (59.4%) (p = 0.02). Similarly, more girls (15.1%) were able to identify iron rich foods than boys (7.7%) (p = 0.03). In Bengaluru, more girls (42.2%) knew about the importance of a balanced diet than boys (22.2%) (p = 0.000). Knowledge on the importance of a balanced diet was more prevalent among private school students than public school students in both cities (p < 0.01). In Bengaluru, more private school students (49.2%) knew salty foods could lead to hypertension, compared to public school students (32.9%) (p = 0.002) ( Table 1).

**Table 1.** Knowledge related to diet and physical activity by gender and school type.

Variable	Overall (N = 1026)	Pune (N = 667)			Bengaluru (N = 359)			Pune (N = 667)			Bengaluru (N = 359)		
	N (%)	Boys N (%)	Girls N (%)	p-value	Boys N (%)	Girls N (%)	p-value	Public School N (%)	Private School N (%)	p-value	Public School N (%)	Private School N (%)	p-value
**Diet**													
Knowledge about balanced diet	262 (25.5)	93 (21.6)	57 (23.9)	0.5	44 (22.2)	68 (42.2)	0.000 **	33 (11.9)	117 (30)	0.000 **	60 (25.9)	52 (40.6)	0.004 **
Should have five servings of fruits and vegetables daily	70 (6.8)	27 (6.3)	12 (5)	0.5	17 (8.6)	14 (8.7)	0.97	11 (4)	28 (7.1)	0.08	19 (8.2)	12 (9.4)	0.710
Eating breakfast daily is important	693 (67.5)	255 (59.4)	162 (68.0)	0.02 *	143 (72.2)	133 (82.6)	0.02 *	178 (64.3)	239 (61.3)	0.434	172 (74.4)	104 (81.2)	0.144
Fat is a calorie dense nutrient	186 (18.1)	81 (18.8)	45 (18.9)	0.99	33 (16.6)	27 (16.7)	0.979	20 (7.2)	106 (27.1)	0.000 **	49 (21.2)	11 (8.5)	0.002 **
Excessive intake of salty foods can lead to hypertension	335 (32.6)	123 (28.7)	73 (30.7)	0.58	78 (39.4)	61 (37.8)	0.771	77 (27.8)	119 (30.5)	0.448	76 (32.9)	63 (49.2)	0.002 **
Sources of iron rich food	101 (9.8)	33 (7.7)	36 (15.1)	0.003 *	21 (10.6)	11 (6.8)	0.212	17 (6.1)	52 (13.3)	0.003 **	15 (6.5)	17 (13.3)	0.03 *
**Physical activity**													
Physical activity decreases the risk of diabetes	681 (66.3)	255 (59.4)	139 (58.4)	0.794	168 (84.8)	119 (73.9)	0.01 *	151 (54.5)	243 (62.3)	0.04 *	196 (84.8)	91 (71.0)	0.002 *
Physical activity decreases the risk of heart diseases	680 (66.2)	264 (61.5)	158 (66.4)	0.213	138 (69.7)	120 (74.5)	0.311	170 (61.3)	252 (64.6)	0.39	173 (74.9)	85 (66.4)	0.08
Physical activity decreases the risk of obesity	714 (69.5)	257 (55.9)	160 (67.2)	0.06	157 (79.3)	140 (86.9)	0.05 *	167 (60.3)	250 (64.1)	0.31	197 (85.3)	100 (78.1)	0.08
60 minutes of physical activity is recommended	194 (18.9)	70 (16.3)	43 (18.0)	0.564	46 (23.2)	35 (21.7)	0.73	47 (16.9)	66 (16.9)	0.988	47 (20.3)	34 (26.5)	0.177
Screen time should be less than 2 hours a day	608 (59.2)	250 (58.2)	154 (64.7)	0.104	102 (51.5)	102 (63.3)	0.02 *	158 (57.0)	246 (63.0)	0.116	124 (53.6)	80 (62.5)	0.106

* P < 0.05.

** p < 0.01.

*Physical activity*

Compared to knowledge about healthy dietary practices, students’ understanding of the benefits of physical activity was higher ( Table 1). Around 66%-69% of students were aware of the positive impact physical activity can have on reducing the risk of NCDs such as diabetes, heart disease, and obesity. Difference in the knowledge level of boys and girls was seen in both Pune and Bengaluru but a significant difference was seen only in Bengaluru for variables linking physical activity with low risk of diabetes (boys: 84.8% vs girls: 73.9%, p = 0.01) and obesity (boys: 79.3% vs girls: 86.9%, p = 0.05). However, less than one-fifth of the students (18.9%, overall) knew that the level of recommended daily physical activity is 60 minutes or more. More students (59.2%, overall) knew screen time should be limited to two hours or less per day.

*Tobacco use*

Few differences in tobacco use knowledge by gender were seen in Pune and Bengaluru. Where present, there was higher comprehension among boys in Pune for items like tobacco use is harmful to health (boys: 83.2% vs girls:76%, p = 0.025) and the legal age for buying or selling tobacco (boys: 61% vs girls: 52.1%, p = 0.025). In Bengaluru, girls had higher knowledge for smoking and its association with heart attack (boys: 84.3% vs girls: 95%, p = 0.001) and cancer (boys: 88.8% , girls: 95%, p = 0.03). In Bengaluru, significantly more public school students were knowledgeable about tobacco use related harms and policies in India than private school students (p < 0.01) ( Table 2).

**Table 2.** Tobacco related knowledge by gender and school type.

Tobacco	Overall (N = 1026)	Pune (N = 667)			Bengaluru (N = 359)			Pune (N = 667)			Bengaluru (N = 359)		
	N (%)	Boys N (%)	Girls N (%)	p-value	Boys N (%)	Girls N (%)	p-value	Public School N (%)	Private School N (%)	p-value	Public School N (%)	Private School N (%)	p-value
All kinds of tobacco use are harmful to health	871 (84.9)	357 (83.2)	181 (76.0)	0.025 *	186 (93.9)	147 (91.3)	0.338	229 (82.6)	309 (79.2)	0.268	220 (95.2)	113 (88.2)	0.01 *
Tobacco use by a young person harms his/her health immediately	679 (66.2)	254 (59.2)	133 (55.8)	0.405	155 (78.3)	137 (85.1)	0.100	149 (53.7)	238 (61.0)	0.062	202 (87.4)	90 (70.3)	0.000 **
Safe to use tobacco products for one or two years	724 (70.5)	272 (63.4)	145 (60.9)	0.526	164 (82.8)	143 (88.8)	0.109	173 (62.4)	244 (62.5)	0.977	212 (91.7)	95 (74.2)	0.000 **
Smoking tobacco (e.g., cigarettes, bidis) causes heart attack	811 (79.0)	321 (74.8)	170 (71.4)	0.340	167 (84.3)	153 (95.0)	0.001 **	207 (74.7)	284 (72.8)	0.582	211 (91.3)	109 (85.1)	0.07
Chewing tobacco (guthka, khaini etc.) causes heart attack	790 (77)	301 (70.1)	181 (76.0)	0.104	170 (85.8)	138 (85.7)	0.969	207 (74.7)	275 (70.5)	0.231	209 (90.4)	99 (77.3)	0.001 **
Smoking tobacco (e.g., cigarettes, bidis) causes stroke	776 (75.6)	308 (71.7)	160 (67.2)	0.217	169 (85.3)	139 (86.3)	0.791	195 (70.4)	273 (70.0)	0.912	212 (91.7)	96 (75.0)	0.000 **
Chewing tobacco (guthka, khaini etc.) causes stroke	765 (74.5)	305 (71.1)	163 (68.4)	0.481	164 (82.8)	133 (82.6)	0.956	189 (68.2)	279 (71.5)	0.358	200 (86.5)	97 (75.7)	0.01 *
Smoking tobacco (e.g., cigarettes, bidis) causes lung cancer	820 (79.9)	314 (73.1)	177 (74.3)	0.741	176 (88.8)	153 (95.0)	0.036 *	203 (73.2)	288 (73.8)	0,871	216 (93.5)	113 (88.2)	0.087
Chewing tobacco (guthka, khaini etc.) causes oral cancer	792 (77.1)	302 (70.4)	163 (68.4)	0.607	176 (88.8)	151 (93.7)	0.105	191 (68.9)	274 (70.2)	0.718	218 (94.3)	109 (85.1)	0.003 **
It’s harmful to your health if you are near a person who is smoking	788 (76.8)	291 (67.8)	165 (69.3)	0.691	181 (91.4)	151 (93.7)	0.396	189 (68.2)	267 (68.4)	0.950	225 (97.4)	107 (83.5)	0.000 **
Quitting tobacco improves a person's health	707 (68.9)	254 (59.2)	137 (57.5)	0.680	177 (89.3)	139 (86.3)	0.375	175 (63.1)	216 (55.3)	0.04 *	218 (94.3)	98 (76.5)	0.000 **
Is there a law which bans sale of tobacco products to and by minors (under 18 years of age)	680 (66.2)	262 (61.0)	124 (52.1)	0.025 *	159 (80.3)	135 (83.8)	0.385	162 (58.4)	224 (57.4)	0.787	203 (87.8)	91 (71.0)	0.000 **
Is there a law which mandates display of a “Tobacco Free School” or “Tobacco Free Institution” sign at a prominent place on the boundary wall outside the main entrance of your school	594 (57.9)	207 (48.2)	117 (49.1)	0.822	140 (70.7)	130 (80.7)	0.028 *	135 (48.7)	189 (48.4)	0.944	209 (90.4)	61 (47.6)	0.000 **
Is there a law which mandates display of a “No smoking area - smoking here is an offence” sign inside your school”?	654 (63.7)	248 (57.8)	128 (53.7)	0.315	160 (80.8)	118 (73.3)	0.090	158 (57.0)	218 (55.9)	0.769	195 (84.4)	83 (64.8)	0.000 **
Is there a law which bans tobacco advertising on television channels and print media?	674 (65.6)	237 (55.2)	120 (50.4)	0.231	176 (88.9)	141 (87.5)	0.701	128 (46.2)	229 (58.7)	0.001 **	216 (93.5)	101 (78.9)	0.000 **
Is there a law which stops people from smoking in public places (like within the premises of your school)?	726 (70.7)	260 (60.6)	150 (63.0)	0.539	172 (86.8)	144 (89.4)	0.455	158 (57.0)	252 (64.6)	0.048 *	216 (93.5)	100 (78.1)	0.000 **

* P < 0.05.

** p < 0.01.

### Attitudes

*Diet*

The maximum score a participant could achieve for attitude towards diet was 40. The mean score of students was 28.4 ± 6.5, which is 70% of the maximum score. Within the two cities, students’ attitude about healthy dietary practices was similar for boys and girls (p > 0.05) and was similar in both school types (p > 0.05) ( Table 3).

**Table 3.** Attitude related to diet, physical activity and tobacco by gender and school type.

Variable		Overall (N = 1026)	Pune (N = 667)			Bengaluru (N = 359)			Pune (N = 667)			Bengaluru (N = 359)		
	Cronbach Alpha	Mean ± SD (95% CI)	Boys Mean ± SD (95% CI)	Girls Mean ± SD (95% CI)	p-value	Boys Mean ± SD (95% CI)	Girls Mean ± SD (95% CI)	p-value	Public School Mean ± SD (95% CI)	Private School Mean ± SD (95% CI)	p-value	Public School Mean ± SD (95% CI)	Private School Mean ± SD (95% CI)	p-value
Diet	0.68	28.4± 6.5 (28.0-28.8)	27.0 ± 7.3 (26.3-27.7)	27.8 ± 7.3 (26.9-28.8)	0.153	30.2 ± 3.8 (29.7-30.7)	30.7±3.8 (30.1-31.3)	0.256	26.7 ± 7 (25.9-27.5)	27.7 ± 7.5 (27-28.5)	0.073	30.2 ± 3.9 (29.7-30.7)	30.8± 3.5 (30.2-31.4)	0.135
Physical activity	0.71	29.5 ± 7.5 (29.1-30.0)	27.4± 8.5 (26.6-28.2)	28.3 ± 7.5 (27.4-29.3)	0.152	32.6 ± 4.5 (31.9-33.2)	33.2 ±4.3 (32.5-33.9)	0.186	26.7 ± 8.3 (25.7-27.7)	28.5 ± 7.9 (27.7-29.3)	0.005 *	33.6 ± 4.5 (33-34.2)	31.5 ± 4.0 (30.8-32.2)	0.001 **
Tobacco use	0.89	31.3 ± 12.7 (30.6-32.1)	26.8 ± 14.1 (25.5-28.2)	29.7 ± 13.3 (28.0-31.4)	0.011 *	37.5 ± 6.5 (36.6-38.4)	38.1±5.7 (37.2-39.0)	0.356	28.3±12.5 (26.8-29.8)	27.6 ±14.8 (26.1-29.0)	0.493	38.5 ± 5.8 (37.8-39.3)	36.4 ± 6.6) (35.2-37.6)	0.002 *

* P < 0.05.

** p < 0.01

SD: Standard deviation, Higher the score better is the attitude towards diet and physical activity and less favourable to tobacco use.

*Physical activity*

The highest score a student could achieve for attitude towards physical activity was 45. The students’ mean score was 29.5 ±7.5, which is 65% of the maximum score. No significant differences were observed between boys and girls in both cities, highlighting that both have a positive attitude towards physical activity (p > 0.05). In Bengaluru, public school students had a more positive attitude towards physical activity in comparison to private school students (p = 0.001), whereas the opposite was observed in Pune ( Table 3).

*Tobacco use*

The maximum score a student could achieve in this construct was 45. The students’ mean score was 31.3 ± 12.7, which is 68% of the maximum score. In Pune, mean scores were significantly higher among girls than boys (29.7 ± 13.3 in girls vs 26.8 ± 14.1 in boys; p = 0.01) and among public school (28.3 ± 12.5) students than private schools’ students (27.6 ± 4.8) (p = 0.01), suggesting girls and public school students had less favourable attitudes towards initiating a tobacco habit. In Bengaluru, no significant differences between gender were observed but a significant difference in scores by school type was observed (public school: 38.5 ± 5.8 vs private school: 36.4 ± 6.6, p = 0.002) ( Table 3).

### Behaviours

*Diet*

Overall, the vast majority of students (94.7%) were eating “outside food” (i.e. takeaway/street food) daily. Few students (8.7%) skipped breakfast daily. In Pune, fewer girls (8.8%) than boys (18.9%) reported that they had never eaten fruits (p = 0.001) or pulses (p = 0.004). In Bengaluru, fewer girls than boys reported that they had never eaten pulses (4.3% girls and 14.1% boys; p = 0.002) or salads (1.2% girls and 11.1% boys; p = 0.000) and more boys (51.8%) than girls (39.4%) reported that they had never read nutrition labels (p = 0.020). In Bengaluru, more girls (46.3%) than boys (35.2%) reported watching television while eating meals (p < 0.01), while the reverse was observed in Pune (boys: 20.2% vs girls 17.2%) (p < 0.01).

With respect to school type, in Pune skipping breakfast and never reading nutrition labels were more common among students in public schools (15.2%) compared to private school (9.7%)(p < 0.05). Nearly one-third of public school students (30%) and 22.7% of private school students never read a food label before buying food items (p < 0.005). Never reading nutrition labels was also very common among public school students in Bengaluru (64.8%) ( Table 4).

**Table 4.** Dietary, physical activity and tobacco use behaviour of students by gender and school type

Behaviours	Overall (N = 1026)	Pune (N = 667)			Bengaluru (N = 359)			Pune (N = 667)			Bengaluru (N = 359)		
	N (%)	Boys N (%)	Girls N (%)	p-value	Boys N (%)	Girls N (%)	p-value	Public School N (%)	Private School N (%)	p-value	Public School N (%)	Private School N (%)	p-value
**Diet**													
Skipping breakfast (daily)	90 (8.7)	56 (13.1)	24 (10.1)	0.258	8 (4.0)	2 (1.2)	0.109	42 (15.2)	38 (9.7)	0.034 *	5 (2.2)	5 (3.9)	0.337
Eat whole fruits in a week (never)	116 (11.3)	81 (18.9)	21 (8.8)	0.001 **	10 (5.1)	4 (2.5)	0.212	39 (14.1)	63 (16.2)	0.463	8 (3.5)	6 (4.7)	0.566
Eat pulses and lentils in a week (never)	167 (16.2)	99 (23.1)	33 (13.9)	0.004 **	28 (14.1)	7 (4.3)	0.002 **	60 (21.7)	72 (18.5)	0.307	23 (10.0)	12 (9.4)	0.859
Eat salads in a week (never)	187 (18.2)	114 (26.6)	49 (20.6)	0.085	22 (11.1)	2 (1.2)	0.000 **	76 (27.4)	87 (22.3)	0.129	16 (6.9)	8 (6.3)	0.806
Eat food/snack while watching TV (always)	261 (26.7)	79 (20.2)	39 (17.2)	0.364	69 (35.2)	74 (46.3)	0.034 *	45 (17.6)	73 (20.1)	0.430	119 (52.2)	24 (18.8)	0.000 **
Read the food labels to choose if the food is healthy or not (never)	311 (33.4)	99 (27.7)	49 (22.4)	0.159	100 (51.8)	63 (39.4)	0.020 *	71 (30.7)	77 (22.3)	0.022 *	147 (64.8)	16 (12.7)	0.000 **
Eat food from outside (daily)	895 (94.7)	334 (92)	218 (95.6)	0.08	189 (97.4)	154 (96.2)	0.527	227 (96.6)	325 (91.3)	0.01 *	220 (96.9)	123 (96.8)	0.973
**Physical activity**													
Screen time (> 2 hours per day)	34 (3.3)	11 (2.6)	5 (2.1)	0.708	13 (6.6)	5 (3.1)	0.135	12 (4.3)	4 (1.0)	0.006 **	16 (6.9)	2 (1.6)	0.026 *
Moderate to vigorous activity less than 60 minutes per day	437 (42.5)	216 (50.3)	124 (52.1)	0.665	52 (26.3)	45 (28.0)	0.720	140 (50.5)	200 (51.3)	0.850	69 (29.9)	28 (21.9)	0.102
**Tobacco**													
Ever tried smoked tobacco (cigarette/beedi/hookah etc.)	289 (30.1)	174 (45.7)	111 (50.2)	0.160	3 (1.5)	1 (0.6)	0.391	174 (65.4)	111 (33.0)	0.001 **	2 (0.9)	2 (1.6)	0.446
Ever tried smokeless tobacco (gutkha, khaini, etc.)	293 (30.5)	179 (46.6)	107 (48.6)	0.347	6 (3.0)	1 (0.6)	0.100	173 (64.8)	113 (33.5)	0.001 **	6 (2.6)	1 (0.8)	0.223
Ever tried electronic vapor product	310 (32.4)	187 (49.3)	118 (53.8)	0.285	3 (1.5)	2 (1.2)	0.827	182 (69.2)	123 (36.7)	0.001 **	2 (0.86)	3 (2.4)	0.243

* P < 0.05.

** p < 0.01.

*Physical activity*

Overall, very few students (3.3%) watched screens (television, computer, video games etc.) for more than two hours a day. No significant differences were seen between girls and boys for screen time either in Pune or Bengaluru (p > 0.05). However, significantly more public-school students compared to private school students reported at least two hours of screen time a day in both Pune (4.3% in public school and 1.0% in private schools; p = 0.006) and Bengaluru (6.9% in public schools and 1.6% in private schools; p = 0.026).

No significant differences in moderate-to-vigorous physical activity were seen by gender or school type in both cities (p > 0.05). However, fewer students in Bengaluru (21.9%-29.9%) reported that they had less than adequate moderate-to-vigorous physical activity per day compared to students in Pune (50.3%-52.1%) ( Table 4).

*Tobacco*

Overall more students in Pune (50.7%) than Bengaluru (2.8%) reported having ever used tobacco. In both cities, no significant differences by gender were observed. However, the prevalence of ever smoking (65.4% vs. 33.0%, p = 0.001), ever use of smokeless tobacco (64.8% vs. 33.5%, p = 0.001), and ever e-cigarette use (69.2% vs. 36.7%, p = 0.001) was significantly higher among public school students in Pune than private school students ( Table 4).

The boys from the participating schools of Pune were found to be more susceptible to tobacco use (23.3% smoking and 25.9% smokeless tobacco) in comparison to the girls (15.5% both smoking and smokeless tobacco use) (p < 0.05) ( Table 5).

**Table 5.** Tobacco use susceptibility of students by gender and school type.

Tobacco	Overall (N = 1026)	Pune (N = 667)			Bengaluru (N = 359)			Pune (N = 667)			Bengaluru (N = 359)		
	N (%)	Boys N (%)	Girls N (%)	p-value	Boys N (%)	Girls N (%)	p-value	Public School N (%)	Private School N (%)	p-value	Public School N (%)	Private School N (%)	p-value
Susceptibility to smokeless tobacco	168 (16.4)	111 (25.9)	37 (15.5)	0.002 **	13 (6.6)	7 (4.3)	0.368	58 (20.9)	90 (23.1)	0.513	11 (4.8)	9 (7.0)	0.369
Susceptibility to smoke tobacco	157 (15.3)	100 (23.3)	37 (15.5)	0.017 **	11 (5.6)	9 (5.6)	0.989	58 (20.9)	79 (20.3)	0.830	15 (6.5)	5 (3.9)	0.306

* P < 0.05.

** p < 0.01.

## Discussion

This is one of the few studies on NCD risk factors that has comprehensively assessed KAB of school going children from two major metropolitan areas of India and the KAB findings helped to develop the school-based health lifestyle programme. During recruitment, it was ensured that the schools included in the study had similar characteristics, as differences in school characteristics might have influenced the results. Given the wide geographic variability in NCDs in this country, ^16, 28, 29^ it is important to assess students’ KAB in a myriad of contexts.

The findings of our study revealed low dietary knowledge among students irrespective of gender and school type. Knowledge was better among girls and private school students. Similar differences were reported in school going students of Haryana (North India). ^30^ The students’ knowledge of physical activity was better than their knowledge of diet and 66%-69% of students were aware of association between NCDs and physical inactivity. In contrast to our study findings, a better knowledge about physical activity (88%) was reported in students of Gujarat, ^31^ and Delhi-India. ^32^ Similarly, adolescents from the eighth and ninth grade in other low-and-middle-income countries (LMICs) such as Nepal, knew that physical activity is imperative for disease prevention and for staying healthy. ^33^

In our study, students’ knowledge about the harms of tobacco use was much higher in comparison to their knowledge about diet and physical activity. This increase in knowledge could be attributed to the Government of India’s efforts to combat rising burden of tobacco use and its associate health impact, including comprehensive national tobacco control law i.e., COTPA-2003, ^34^ National Tobacco Control Programme ^35^ as well as tobacco-free educational institutional guidelines. ^36^ Evidence from other LMICs, including Bangladesh, also reported high awareness of the harms of tobacco use among rural adolescents (13-18 years). ^37^

With regard to behaviours, only 8.7% of students were missing their breakfast daily. This is similar to the findings of a study conducted among school students of Gujarat, India. ^38^ Findings from a study conducted in Kenya also showed similar results, wherein 92.1% of the children and adolescents (8-11 years) consumed breakfast daily. ^39^ In our study, more students from public schools reported missing their daily breakfast than students in private schools. This could be attributed to a low level of knowledge and the presence of students from lower middle-income families in these schools. Students from lower income families are more likely to experience scarcity of good quality food, meaning they may lack a wholesome breakfast as well as other meals of the day. ^40^ An additional factor could be the availability of a mid-day meal scheme (i.e., school meal) in public schools, ^41^ which may preclude the importance of their first meal at home (i.e., breakfast).

Other protective dietary factors include fruit consumption, and only 11.3% of students did not eat fruits daily. The Comprehensive National Nutrition Survey (2019) showed that among adolescents (10-14 years), 59% in Maharashtra and 74% in Karnataka ate whole fruits only once a week. ^28^ Though fruits are “price elastic” (a term that describes the connection between income and the demand of a product), ^42, 43^ no difference in consumption was seen by school type. Results from other LMICs were poorer, with only 9% of adolescents (8-11 years) consuming fruits four to seven times a week. ^39^ On other hand, in a cross-country comparison among 49 LMICs, India had highest percentage (29.5%) of adolescents (13-17 years old) who met WHO’s recommendation for fruit and vegetable consumption. ^44^ All of the included 49 countries were selected due to the availability of the Global School Based Student Health Survey (GSHS) data from 2004-2013 for this ecological study and provided a data on a total of 164,771 adolescents.

Another worrisome behaviour observed among students was the habit of eating “outside food” (i.e., takeout) every day (95%). Contributing factors to its popularity could be ease of access, taste, parent’s occupation, and fast-food companies marketing strategies. ^45^ Fast food consumption data from 153,496 young adolescents (12-15 years) from 54 LMICs showed that 55.2% (51.3–59.1%) of the adolescents consumed fast food at least once per week, and 10.3% (8.3–12.4%) consumed fast food between four to seven days per week. The prevalence of fast-food consumption for four to seven days per week was lowest in the Americas (8.3%; 95% CI: 6.7–9.9) and highest in Southeast Asia (17.7%; 95% CI: 2.3–33.2%). ^46^ Our study found that students are not reading nutritional labels while making food choices, as only 33% of students read labels. Students in public schools were practicing this behaviour more often than students in private schools. In contrast to this trend, 88% of students in Kolkata reported reading nutritional labels, and this was most common among girls. ^47^

Lack of physical activity among adolescents is a major NCD risk factor. More than half of the students (57.5%) in this study reported engaging in moderate-to-vigorous physical activity for at least 60 minutes, daily, with no significant differences by gender or school type. One recent publication showed that 73.9% of adolescents in India are insufficiently physically active. ^48^ However, a global study of 49 LMICs with 164,771 adolescents found that India had the highest percentage (29.5%) of adolescents who met WHO’s recommendations for physical activity. ^44^ On an encouraging note, in the same study, few adolescents (3%) reported screen time of more than two hours a day. This is in contrast to 52.5% of school going adolescents in Tamil Nadu (Southmost part of India) who had an average daily screen time of more than two hours a day. ^49^

One third of adolescents had ever tried tobacco (smoke, smokeless and e-cigarette). Evidence from 68 LMICs showed that mean prevalence of tobacco use among adolescents (12-15 years) was 13.6%, ranging from 2·8% in Tajikistan to 44·7% in Samoa and in most countries prevalence among boys was higher compared to girls. ^50^ However, no difference among boys and girls was observed in this study, indicating a narrowing of gender gap for tobacco use which is much wider among adults in India. ^51^ In Pune, public school students used more tobacco (all forms) in comparison to private schools. Whereas in Bengaluru, smokeless tobacco was more commonly used among public school students in comparison to smoke forms. Tobacco use (all forms) was found to be more prevalent in public school boys (13.4%) in comparison to private school boys (11.7%). ^52^ This could be due to the students’ lack of compliance with provisions of Indian tobacco control law as well as the availability and ease of access to tobacco products outside of their schools. ^53^ Surprisingly, 32.4% of the students also reported that they have tried e-cigarettes in the past. To the best of our knowledge, no other study in India has yet reported the prevalence of e-cigarette use among school going children. The prevalence found in our study was much higher than that found in a study from Mexico. ^54^

Results suggest that tobacco use prevention will be especially important for schools in Pune, while promoting physical activity may be a key strategy to pursue in Bengaluru. Students in both cities would benefit from additional school-based intervention to support healthy dietary practices, including eating pulses and vegetables frequently; eating healthier, eating home-cooked meals; and reading nutrition labels.

As the 2030 deadline for Sustainable Development Goals are only a decade away, the low levels of knowledge, particularly about unhealthy diet and physical inactivity, are a matter of concern. ^55^ Aiming to reduce the NCD burden, the budding role of education in primary prevention was emphasised at the 2011 United Nations’ Meeting on the Prevention and Control of NCDs. ^56^ Lack of healthy behaviours among adolescents has been linked with lack of sufficient knowledge, and research has also indicated a positive relationship between information level and overt behaviour. ^57^ Sufficiently designed health promotion programmes ^58^ may provide the much needed knowledge that will help to reduce risk behaviours such as unhealthy diet, physical inactivity, and tobacco use, hence providing a pathway for behaviour change. Health promotion strategies in schools provide an avenue to reach school children at a time coinciding with their cognitive development. Such an approach can impart information effectively, especially with respect to lifestyle choices. ^59^ Studies have shown that if implemented efficiently, health promoting schools can enhance decision-making skills among students, too. ^60^

In India, various education programmes have been designed for school going adolescents but there are only few comprehensive education programs addressing diet, physical activity and tobacco. ^61^ In 2018, the Government of India launched the National School Health Programme under Ayushman Bharat or Healthy India. The programme comprehensively addresses the above risk factors, and is delivered by health ambassadors (two teachers per school) only in government and government aided schools across country. ^62^

The results of the present study are being used to inform the development of a two-year intervention programme in these cities which will target students in the sixth to eighth grades, their parents, and community members. This intervention programme is being implemented from 2019-2021 by trained teachers and is facilitated by trained peer leaders in both public and private schools. This model of delivery has proven successful in India and elsewhere globally for tobacco ^63^ and nutrition ^12^ education.

One of the limitations of this study was that the sample of schools was not randomly selected from the population but was representative of the mix of types of schools in these two cities. This limits generalisability of the findings. The self-reported method utilised (i.e., survey) also may have led to skewed estimates of dietary intake, physical activity and tobacco use patterns among the students due to the fact that adolescents would have wanted to report positive health behaviours (social desirability - reporting bias). Behaviour for every type of tobacco use was evaluated without further investigating the number of times the tobacco was used by the students. Given budgetary constraints, no anthropometric or biochemical (e.g., salivary cotinine) measurements were completed, which may be a drawback of this study.

## Conclusion

There is a need to amplify health education activities in schools in India, and school health interventions need to be adequately designed to ensure that they do not further widen existing socio-economic inequalities. This study highlights the need to augment school health programmes in India with a differential approach based on the issues which are specific to each school type (public and private) and city (Pune and Bengaluru). School health programmes should be structured with a dedicated focus on health promotion and NCD prevention. Developed intervention materials should be incorporated in the curriculum to become part of the regular teaching.

## Data availability

### Underlying data

Figshare: Knowledge, Attitude and Behaviours (KAB) on diet, physical activity, and tobacco use among school students: Survey Dataset. https://doi.org/10.6084/m9.figshare.14760147.v4. ^64^

This project contains the following underlying data:

• KAB Survey Dataset.xlsx (student knowledge, attitude and behaviour responses regarding their diet, physical activity and tobacco consumption).

### Extended data

Figshare: Knowledge, Attitude and Behaviours (KAB) on diet, physical activity, and tobacco use among school students: Survey Tool. https://doi.org/10.6084/m9.figshare.14760480.v2. ^65^

This project contains the following extended data:

• KAB Questionnaire_English.pdf
• KAB Questionnaire_Marathi.pdf
• KAB Questionnaire_Kannada.pdf

Data are available under the terms of the Creative Commons Attribution 4.0 International license (CC-BY 4.0).

## References

[1] World Health Organization: Non-communicable diseases: progress monitor 2020. Geneva: World Health;2020.

[2] World Health Organisation: Global Status Report on Non-Communicable Diseases. 2011. Reference Source

[3] Institute for Health Metrics and Evaluation: Global Health Data. GBD Results Tool|GHDx. Reference Source

[4] WHO: Global action plan for the prevention and control of noncommunicable diseases 2013-2020. *World Heal Organisation.* 2013;102. Reference Source

[5] Watkins D Hale J Hutchinson B : Investing in non-communicable disease risk factor control among adolescents worldwide: A modelling study. *BMJ Glob Heal.* 2019 Apr 1;4(2). 10.1136/bmjgh-2018-001335 31139451 PMC6509594

[6] Paavola M Vartiainen E Haukkala A : Smoking, alcohol use, and physical activity: A 13-year longitudinal study ranging from adolescence into adulthood. *J Adolesc Heal.* 2004 Sep;35(3):238–244. 10.1016/j.jadohealth.2003.12.004 15313507

[7] Craigie AM Lake AA Kelly SA : Tracking of obesity-related behaviours from childhood to adulthood: A systematic review. *Maturitas.* Maturitas;2011;70: p.266–84. 10.1016/j.maturitas.2011.08.005 21920682

[8] Sawyer SM Afifi RA Bearinger LH : Adolescence: A foundation for future health. *Lancet.* Lancet Publishing Group;2012;379: p.1630–40. 10.1016/S0140-6736(12)60072-5 22538178

[9] World Health Organization Regional Office for South-East Asia: Strategic Guidance on Accelerating Actions for Adolescent Health. 2018.

[10] Cilluffo A Ruiz NG : World’s population is projected to nearly stop growing by the end of the century. Pew Research Center;2019. Reference Source

[11] Singh A Bassi S Nazar GP : Impact of school policies on non-communicable disease risk factors - a systematic review. *BMC Public Health.* 2017 Apr 4;17(1):292. Reference Source 28376833 10.1186/s12889-017-4201-3 PMC5379668

[12] Bassi S Gupta VK Chopra I : Novel school-based health intervention program—a step toward early diabetes prevention. *Int J Diabetes Dev Ctries.* 2015 Nov 1;35(4):460–468. Reference Source

[13] Langford R Bonell CP Jones HE : The WHO Health Promoting School framework for improving the health and well-being of students and their academic achievement. *Cochrane Database of Systematic Reviews.* John Wiley and Sons Ltd;2014;2014. 10.1002/14651858.CD008958.pub2 PMC11214127 24737131

[14] Public Health Foundation of India: Project PaTHWay: PromoTing Health and Wellbeing. 2019. Reference Source

[15] Express Healthcare: Directorate of Health Services, Maharashtra and PHFI collaborate for Project PaTHWay. 2019; Reference Source

[16] Indian Council of Medical Research, Public Health Foundation of India, Institute for Health Metrics and Evaluation: GBD India Compare. 2016. Reference Source

[17] Directorate of Census Operations: District Census Handbook, Pune Maharashtra: Census of India. Pune;2011. Reference Source

[18] Kumar TKAK : Directorate of Census Operations Karnataka. *CENSUS OF INDIA 2011: Primary Census Abstract Data Highlights Karnataka.* 2011.

[19] Bandura A : Health promotion by social cognitive means. *Health Educ Behav.* 2004;31: p.143–64. 10.1177/1090198104263660 15090118

[20] Global Youth Tobacco Survey Collaborative Group: *Global Youth Tobacco Survey (GYTS): Core Questionnaire with Optional Questions* . GA, Atlanta;2014.

[21] Global Adult Tobacco Survey Collaborative Group: Global Adult Tobacco Survey (GATS): Core Questionnaire with Optional Questions. *Centers Dis Control Prev.* 2010; Reference Source

[22] Neumark-Sztainer D Story M Hannan PJ : Overweight status and eating patterns among adolescents: Where do youths stand in comparison with the Healthy People 2010 objectives? *Am J Public Health.* 2002;92(5):844–51. 10.2105/ajph.92.5.844 11988458 PMC1447172

[23] Hoelscher DM Day RS Kelder SH : Reproducibility and validity of the secondary level School-Based Nutrition Monitoring student questionnaire. *J Am Diet Assoc.* 2003;103(2):186–194. 10.1053/jada.2003.50031 12589324

[24] Reddy KS Arora M Kohli A : Tobacco and Alcohol Use Outcomes of a School-based Intervention in New Delhi. *Am J Health Behav.* 2002 May 1;26(3):173–181. 10.5993/ajhb.26.3.2 Reference Source 12018753

[25] Perry CL Stigler MH Arora M : Preventing tobacco use among young people in India: Project MYTRI. *Am J Public Health.* 2009;99(5):899–906. 10.2105/AJPH.2008.145433 19299670 PMC2667859

[26] Bassi S Gupta VK Park M : School policies, built environment and practices for non-communicable disease (NCD) prevention and control in schools of Delhi, India. Tadakamadla SK , editor. *PLoS One.* 2019 Apr 18;14(4):e0215365. 10.1371/journal.pone.0215365 30998714 PMC6472740

[27] StataCorp: Stata Statistical Software. Release 12. College Station, TX: StataCorp LP.;2013.

[28] Ministry of Health and Family Welfare Government of India: Comprehensive National Nutrition Survey. 2016-2018.2018. Reference Source

[29] Gulati A Hochdorn A Paramesh H : Physical Activity Patterns Among School Children in India. *Indian J Pediatr.* Springer;2014;81: p.47–54. 10.1007/s12098-014-1472-x Reference Source 24944142

[30] Rani J : Impact of Nutritional Knowledge Status of Adolescents on their Health [Internet]. *Int J Innovations Engineering Technology (IJIET).* 2013;3. Reference Source

[31] Puwar T Saxena D Yasobant S : Noncommunicable diseases among school-going adolescents: A case study on prevalence of risk factors from Sabarkantha District of Gujarat, India. *Indian J Community Med.* 2018;43(5):33. 10.4103/ijcm.IJCM_117_18 Reference Source 30686872 PMC6324039

[32] Yadav S Khokhar A : Effect of information, education, and communication activity on health literacy of obesity and physical activity among school-going adolescents in Delhi. *Indian J Community Fam Med.* 2020;6(1):22. 10.4103/jehp.jehp_756_19 Reference Source 34084818 PMC8057185

[33] Pahlm J Svensson J Joshi SK : Physical activity and diet among adolescents of Kathmandu, Nepal: Knowledge and attitudes. *J Kathmandu Med Coll.* 2014 Jun 18;2(2):51–58. 10.3126/jkmc.v2i2.10623

[34] National Health Mission, Government of India: COTPA 2003 and Rules made thereunder. Reference Source

[35] Ministry of Health and Family Welfare Government of India: National Tobacco Control Programme: Operational Guidelines. New Delhi;2015. Reference Source

[36] Ministry of Health and Family Welfare Government of India: Guidelines for Tobacco Free Educational Institution (Revised). New Delhi;2018.

[37] Ullah MZ Lim JNW Ha MA : Smokeless tobacco use: Pattern of use, knowledge and perceptions among rural Bangladeshi adolescents. *PeerJ.* 2018 Aug 21;2018(8):e5463. 10.7717/peerj.5463 Reference Source 30155362 PMC6108312

[38] Kotecha PV Patel SV Baxi RK : Dietary pattern of schoolgoing adolescents in Urban Baroda, India. *J Heal Popul Nutr.* 2013 Dec;31(4):490–496. 10.3329/jhpn.v31i4.20047 24592590 PMC3905643

[39] Kigaru DMD Loechl C Moleah T : Nutrition knowledge, attitude and practices among urban primary school children in Nairobi City. *Kenya: a KAP study. BMC Nutr.* 2015 Dec 29;1(1):1–8. 10.1186/s40795-015-0040-8

[40] Arora M Nazar GP Gupta VK : Association of breakfast intake with obesity, dietary and physical activity behavior among urban school-aged adolescents in Delhi, India: Results of a cross-sectional study. *BMC Public Health.* 2012;12(1). 10.1186/1471-2458-12-881 23075030 PMC3549919

[41] Ministry of Human Resource Development, Government of India: Mid Day Meal Scheme. Reference Source

[42] Andreyeva T Long MW Brownell KD : The impact of food prices on consumption: A systematic review of research on the price elasticity of demand for food. *Am J Public Health.* American Public Health Association;2010;100: p.216–22. 10.2105/AJPH.2008.151415 20019319 PMC2804646

[43] Kavitha V Umanath M Paramasivam R : Determinants of Consumption Probability and Demand for Fruits in India. *Agric Econ Res Rev.* 2016;29(conf):161. 10.5958/0974-0279.2016.00043.4

[44] Darfour-Oduro SA Buchner DM Andrade JE : A comparative study of fruit and vegetable consumption and physical activity among adolescents in 49 Low-and-Middle-Income Countries. *Sci Rep.* 2018 Dec 1;8(1):1–12. 10.1038/s41598-018-19956-0 Reference Source 29374197 PMC5785955

[45] Das JC : Fast Food Consumption in Children: A Review; Reference Source

[46] Li L Sun N Zhang L : Fast food consumption among young adolescents aged 12–15 years in 54 low- and middle-income countries. *Glob Health Action.* 2020 Dec 31;13(1):1795438–1795438. 10.1080/16549716.2020.1795438 32762333 PMC7480506

[47] Saha S Vemula SR Mendu VVR : Knowledge and practices of using food label information among adolescents attending schools in Kolkata, India. *J Nutr Educ Behav.* 2013 Nov 1;45(6):773–779. 10.1016/j.jneb.2013.07.011 24021455

[48] Guthold R Stevens GA Riley LM : Global trends in insufficient physical activity among adolescents: a pooled analysis of 298 population-based surveys with 1·6 million participants. *Lancet Child Adolesc Heal.* 2020 Jan 1;4(1):23–35. 10.1016/S2352-4642(19)30323-2 31761562 PMC6919336

[49] Kumar SS Shirley SA : Association of screen time with physical activity and BMI in middle school children at Tamil Nadu, India. *Int J Contemp Pediatr.* 2019 Dec 24;7(1):78. 10.18203/2349-3291.ijcp20195730 Reference Source

[50] Xi B Liang Y Liu Y : Tobacco use and second-hand smoke exposure in young adolescents aged 12-15 years: data from 68 low-income and middle-income countries. *Lancet Glob Heal.* 2016;4(11):e795–e805. 10.1016/S2214-109X(16)30187-5 Reference Source 27697484

[51] Ng M Freeman MK Fleming TD : Smoking prevalence and cigarette consumption in 187 countries, 1980-2012. *JAMA - J Am Med Assoc.* 2014 Jan 8;311(2):183–192. 10.1001/jama.2013.284692 Reference Source 24399557

[52] Narain R Sardana S Gupta S : Age at initiation & prevalence of tobacco use among school children in Noida, India: A cross-sectional questionnaire based survey. *Indian J Med Res.* 2011 Mar;133(3):300–307. 21441684 PMC3103155

[53] John Hopkins Bloomberg School of Public Health, Milken Institute of Public Health: Compliance with the Cigarette and Other Tobacco Products Act (COTPA) Results from 2012 and 2013: Maharashtra. Reference Source

[54] Lozano P Barrientos-Gutierrez I Arillo-Santillan E : A longitudinal study of electronic cigarette use and onset of conventional cigarette smoking and marijuana use among Mexican adolescents. *Drug Alcohol Depend.* 2017 Nov 1;180:427–430. 10.1016/j.drugalcdep.2017.09.001 28988005 PMC5771440

[55] United Nations: Sustainable Development Goals. 2015. Reference Source

[56] Bay JL Hipkins R Siddiqi K : School-based primary NCD risk reduction: education and public health perspectives. *Health Promot Int.* 2017;32:369–379. 10.1093/heapro/daw096 Reference Source 28011654

[57] Fleary SA Joseph P Pappagianopoulos JE : Adolescent health literacy and health behaviors: A systematic review. *J Adolesc.* Academic Press;2018;62: p.116–127. 10.1016/j.adolescence.2017.11.010 29179126

[58] Magarey AM Pettman TL Wilson A : Changes in Primary School Children’s Behaviour, Knowledge, Attitudes, and Environments Related to Nutrition and Physical Activity. *ISRN Obes.* 2013;2013:1–10. 10.1155/2013/752081 24555153 PMC3901960

[59] Day RE Sahota P Christian MS : Effective implementation of primary school-based healthy lifestyle programmes: A qualitative study of views of school staff. *BMC Public Health.* 2019 Sep 9;19(1):1239. 10.1186/s12889-019-7550-2 Reference Source 31500603 PMC6734437

[60] Karkada S Pai MS : Concept of Health Promoting School. *Manipal J Nurs Heal Sci.* 2016;2(2):65–8. Reference Source

[61] Saraf DS Gupta SK Pandav CS : Effectiveness of a School Based Intervention for Prevention of Non-communicable Diseases in Middle School Children of Rural North India: A Randomized Controlled Trial. *Indian J Pediatr.* 2015;82(4):354–362 10.1007/s12098-014-1562-9 25209052

[62] Ministry of Health and Family Welfare and Ministry of Human Resource Development: School Health Programme under Ayushman Bharat, Operational Guidelines. New Delhi;2018. Reference Source

[63] Arora M Stigler MH Reddy KS : Effectiveness of health promotion in preventing tobacco use among adolescents in India research evidence informs the national tobacco control programme in India. *Glob Health Promot.* 2011;18(1):09–12. 10.1177/1757975910393163 21721292 PMC3132087

[64] Bassi S : Knowledge, Attitude and Behaviours (KAB) on diet, physical activity, and tobacco use among school students: Survey Dataset. *figshare. Dataset.* 2021. 10.6084/m9.figshare.14760147.v4 PMC8543165 34745560

[65] Bassi S : Knowledge, Attitude and Behaviours (KAB) on diet, physical activity, and tobacco use among school students: Survey Tool. *figshare. Dataset.* 2021. 10.6084/m9.figshare.14760480.v2 PMC8543165 34745560

