# Supplementary material for: Knowledge, attitude, and behaviours on diet, physical activity, and tobacco use among school students: A cross-sectional study in two Indian states
Source: F1000Res. 2021 Oct 15;10:544. Originally published 2021 Jul 7. [Version 2] doi: 10.12688/f1000research.51136.2 (PMC8543165; doi:10.12688/f1000research.51136.2)
Supplement: Peer review history

# Peer review: Reviewer response for version 2

**DOI:** 10.5256/f1000research.77935.r97069

**Published:** 2021-10-22

**Authors:** Subhash Pokhrel

**Affiliations:** 1 Health Economics Research Group, Division of Global Public Health, Department of Health Sciences, Brunel University London, Uxbridge, UK

The manuscript now addresses my previous concerns.

Is the work clearly and accurately presented and does it cite the current literature?

Yes

If applicable, is the statistical analysis and its interpretation appropriate?

Partly

Are all the source data underlying the results available to ensure full reproducibility?

Yes

Is the study design appropriate and is the work technically sound?

Yes

Are the conclusions drawn adequately supported by the results?

Partly

Are sufficient details of methods and analysis provided to allow replication by others?

Yes

Reviewer Expertise:

Public health, behaviour change, health economics

I confirm that I have read this submission and believe that I have an appropriate level of expertise to confirm that it is of an acceptable scientific standard.

# Peer review: Reviewer response for version 1

**DOI:** 10.5256/f1000research.54264.r91108

**Published:** 2021-08-23

**Authors:** Nishibha Thapliyal

**Affiliations:** 1 Department of Sports Science, Punjabi University Patiala, Patiala, Punjab, India

The paper **“**Knowledge, attitude and behaviours on diet, physical activity and tobacco use among school students: A cross-sectional study in two Indian states by Shalini Bassi *et.al*. looks at measuring the knowledge, attitudes and behaviours (KAB) of school children (grade 6) in relation to NCD behavioural risk factors, primarily unhealthy dietary intake, physical inactivity and tobacco use. This information gathered is the baseline evaluation of Project PaTHway (a 3-year school-based healthy lifestyle programme).

           

 Overall, an interesting and impressive read. The strength of the paper lies in its massive contribution (KAB findings) towards limited literature of school-going children about NCDs awareness and also in providing emphasis to support the development of school-based health education programmes (policy) in India. However, since the subject population from rural areas (villages) could not be captured, it is suggested to be added as a limitation.

**Abstract:  **It is an extremely well structured abstract with reasonable focus on the key areas.

**Introduction: **The introduction is sound and clearly demarcates the problem that will be addressed in the paper. The area of focus and the aims are easily identified. However, a little more on the prospective role of KAB as a tool to develop any kind of intervention programme can be added. A little on the current behavioural habits of school children can also be mentioned, including the reason for the selection of school children (specifically 6 ^th^ grade) as the study population.

**Methods: **It is a well planned and thoroughly designed methodology, keeping in mind all permissions, clearance and consents requirement of a good study design. The credibility and validity of the tools have also been established. However, certain factors that could not be controlled (home environment) should be mentioned as a limitation. Also, the use of an additional supportive tool (like a food frequency or anthropometric) to decrease the reporting bias of a ‘self-reporting survey’ (also highlighted by the author) should be mentioned as a suggestive approach for future researches.

**Results & Discussion: **The results are extensive and logically sequenced with a simple and easy to understand presentation. All areas of interest (KAP of dietary intake, tobacco use and physical inactivity) have been clearly depicted. However, there are certain findings (results of fruit intake, screen time and consumption of outside food) that challenge the traditional findings/beliefs of people’s behaviours that vary due to socioeconomic inequality. This could be due to the bias (subjects giving out socially approved answers) in self-reporting surveys (also listed as a limitation by the author) and can be added as a limitation in the study.

**Conclusion:** The findings of this project correctly identify the need for school-based lifestyle intervention programmes in terms of national policy planning for the prevention of NCD’s. The conclusion is precise and clear.

Is the work clearly and accurately presented and does it cite the current literature?

Yes

If applicable, is the statistical analysis and its interpretation appropriate?

Yes

Are all the source data underlying the results available to ensure full reproducibility?

Yes

Is the study design appropriate and is the work technically sound?

Yes

Are the conclusions drawn adequately supported by the results?

Yes

Are sufficient details of methods and analysis provided to allow replication by others?

Yes

Reviewer Expertise:

Public health nutrition, physical activity, NCD, obesity

I confirm that I have read this submission and believe that I have an appropriate level of expertise to confirm that it is of an acceptable scientific standard.

# Response

**Published:** 2021-09-23

**Authors:** Shalini Bassi

**Affiliations:** Public Health Foundation of India, India

Overall, an interesting and impressive read. The strength of the paper lies in its massive contribution (KAB findings) towards limited literature of school-going children about NCDs awareness and also in providing emphasis to support the development of school-based health education programmes (policy) in India. However, since the subject population from rural areas (villages) could not be captured, it is suggested to be added as a limitation.

- **Response:** Suggestion accepted. Added a line in the limitation section.

**Abstract: **It is an extremely well structured abstract with reasonable focus on the key areas.

**Introduction: **The introduction is sound and clearly demarcates the problem that will be addressed in the paper. The area of focus and the aims are easily identified. However, a little more on the prospective role of KAB as a tool to develop any kind of intervention programme can be added. A little on the current behavioural habits of school children can also be mentioned, including the reason for the selection of school children (specifically 6 ^th^ grade) as the study population.

- **Response**: Suggestion accepted, we have incorporated a justification for assessing a KAB at baseline. “ *The baseline analysis of the KAB of the target population would provide better insight on the ‘on-ground’ situation and can guide the development of the intervention programme*”.
- We have included a justification of recruiting class 6th students under the Introduction section: “ *Recruitment of sixth graders was done as the World Health Organization defines that adolescent age begins at 10 years. When a child enters adolescence, there is a change in explorative and emotive behaviors due to the shift from parental dependence to independence. Adolescents are vulnerable as they gain more control of what, when, how, and where they eat; moreover, their preferences and personal choices may gain priority over eating habits that are nurtured within the family setting*”.

**Methods: **It is a well-planned and thoroughly designed methodology, keeping in mind all permissions, clearance and consents requirement of a good study design. The credibility and validity of the tools have also been established. However, certain factors that could not be controlled (home environment) should be mentioned as a limitation. Also, the use of an additional supportive tool (like a food frequency or anthropometric) to decrease the reporting bias of a ‘self-reporting survey’ (also highlighted by the author) should be mentioned as a suggestive approach for future researches.

- **Response: **With context to the role of home environment in shaping the individual behavior has been added in the limitation section. We have also added the use of a food frequency questionnaire in the limitation section.

**Results & Discussion: **The results are extensive and logically sequenced with a simple and easy to understand presentation. All areas of interest (KAP of dietary intake, tobacco use and physical inactivity) have been clearly depicted. However, there are certain findings (results of fruit intake, screen time and consumption of outside food) that challenge the traditional findings/beliefs of people’s behaviours that vary due to socioeconomic inequality. This could be due to the bias (subjects giving out socially approved answers) in self-reporting surveys (also listed as a limitation by the author) and can be added as a limitation in the study.

- **Response: **This information already exists in the limitation section: “The self-reported method utilised (i.e., survey) also may have led to skewed estimates of dietary intake, physical activity and tobacco use patterns among the students due to the fact that adolescents would have wanted to report positive health behaviours (social desirability - reporting bias).”.

**Conclusion:** The findings of this project correctly identify the need for school-based lifestyle intervention programmes in terms of national policy planning for the prevention of NCD’s. The conclusion is precise and clear.

- **Response: **Thanks.

# Peer review: Reviewer response for version 1

**DOI:** 10.5256/f1000research.54264.r89265

**Published:** 2021-08-17

**Authors:** Subhash Pokhrel

**Affiliations:** 1 Health Economics Research Group, Division of Global Public Health, Department of Health Sciences, Brunel University London, Uxbridge, UK

This is a well-written paper that provides data on the knowledge, attitude and behaviours of school students in two Indian states. Such data will provide a baseline against which the impact of any behaviour change intervention can be measured in the future. As such, this is a timely and valuable paper.

 A few areas where the authors may want to improve on:

**Abstract**

1. Conclusions: The last few sentences are policy speculations, not study conclusions, so they may be better placed in the Discussion section instead (of course with some backup references there).

**Methods**

1. Setting: Purposive sampling technique - explain a bit further to explain how exactly the 20 schools were selected. What was the sampling frame (total number of schools) and how exactly did you balance various characteristics mentioned here.
2. Participants: The number given for the final sample size (n=1238) does not match the number given in the Results section. Need to rephrase this sentence.
3. Statistical analysis: A commentary needs to be added in the limitation section later saying that statistical tests are indicative only, as the sampling design was purposive (not random). Also, need justification for a t-test in the light of Likert-scale data.

**Results**

1. The presentation looks fine as this is largely descriptive data. However, I wonder whether additional analysis examining if the reported KAB differed by parental education and occupation may provide more helpful insights. In other words, would observed gender differences in KAB disappear (or the other way around) once you have controlled for parental education and/or parental occupation? Note that in the Discussion section (p.13, para 6), you hypothesise the habit of eating outside food may have been influenced by parental occupation, among other things. Why not test these when you have collected your own data?

**Discussion**

1. Most space in the Discussion section has been devoted to compare and contrast study findings with wider literature, which is important but doing so has limited the ability for the study to provide valuable insights for policymakers in relation to answering “what next”. So, you may choose to summarise the comparison of your findings with the study results in addition to providing a succinct discussion as to how Indian (and wider South Asian) policymakers can go from here. For example, for the sake of the argument, if we followed COM-B model of behaviour change to design and implement interventions for this age group, how can the study results help us identify where capabilities, opportunities and motivations lie and how can adolescents be supported in their lacking space to change their behaviour. I think the rich discussion in this area makes this paper more useful.
2. A confusion/error, p.13, para 5: Seems to be a confusion between “price elasticity” and “income elasticity”. Please revise the sentence.
3. Limitations: Acknowledge that statistical tests are indicative only as the whole sample was purposive.

**Conclusion**

1. Strongly recommend revising the conclusion section. The sentences here must be backed up by the study findings and leave any speculative policies to the Discussion section above. An example conclusion may be: “In the two states of India, a higher proportion of school students know the harm of tobacco use than the benefits of a healthy diet and physical activity. Both gender of the student and which type of the school that the student went to influenced specific KABs but both the magnitude and direction of such influence were variable. The data suggests that there is an urgent need for designing and implementing interventions that can improve school students’ capability and motivation as well as create opportunities to change their unhealthy behaviours.

Is the work clearly and accurately presented and does it cite the current literature?

Yes

If applicable, is the statistical analysis and its interpretation appropriate?

Partly

Are all the source data underlying the results available to ensure full reproducibility?

Yes

Is the study design appropriate and is the work technically sound?

Yes

Are the conclusions drawn adequately supported by the results?

Partly

Are sufficient details of methods and analysis provided to allow replication by others?

Yes

Reviewer Expertise:

Public health, behaviour change, health economics

I confirm that I have read this submission and believe that I have an appropriate level of expertise to confirm that it is of an acceptable scientific standard, however I have significant reservations, as outlined above.

# Response

**Published:** 2021-09-23

**Authors:** Shalini Bassi

**Affiliations:** Public Health Foundation of India, India

**Abstract**

1. Conclusions: The last few sentences are policy speculations, not study conclusions, so they may be better placed in the Discussion section instead (of course with some backup references there).
   
  - **Response:** This has been revised as suggested. The conclusion highlighted the key study findings.

 
**Methods**

1. Setting: Purposive sampling technique - explain a bit further to explain how exactly the 20 schools were selected. What was the sampling frame (total number of schools) and how exactly did you balance various characteristics mentioned here.
   
  - **Response**: Schools and colleges were selected based on the existing network of collaboration to represent different socio-economic statuses. This information has been incorporated under the setting section to make information comprehendible.
2. Participants: The number given for the final sample size (n=1238) does not match the number given in the Results section. Need to rephrase this sentence.
   
  - **Response**: The line “Total sample of 1238 students (Pune: n = 806; Bengaluru: n = 432) were included in the present study” has been deleted. The existing lines under this section emphasized that 1238 students were eligible and invited to participate in the study. Out of 1238 eligible students, 1026 students participated, which is already highlighted in the result section.
3. Statistical analysis: A commentary needs to be added in the limitation section later saying that statistical tests are indicative only, as the sampling design was purposive (not random). Also, need justification for a t-test in the light of Likert-scale data.
   
  - **Response**: Suggested accepted, added a line in the limitation section regarding purposive sampling. Each attitude statement was given a score and a mean score was calculated for this construct. This has been explained under the instrument and measure section. Thus, a t-test was used for this construct to do gender and school type comparisons within each city.

**Results**

1. The presentation looks fine as this is largely descriptive data. However, I wonder whether additional analysis examining if the reported KAB differed by parental education and occupation may provide more helpful insights. In other words, would observed gender differences in KAB disappear (or the other way around) once you have controlled for parental education and/or parental occupation? Note that in the Discussion section (p.13, para 6), you hypothesise the habit of eating outside food may have been influenced by parental occupation, among other things. Why not test these when you have collected your own data?
   
  - **Response:** We anticipated doing this analysis, however, many of the students did not attempt the questions (missing response) related to their parental education and occupation.

**Discussion**

1. Most space in the Discussion section has been devoted to compare and contrast study findings with wider literature, which is important but doing so has limited the ability for the study to provide valuable insights for policymakers in relation to answering “what next”. So, you may choose to summarise the comparison of your findings with the study results in addition to providing a succinct discussion as to how Indian (and wider South Asian) policymakers can go from here. For example, for the sake of the argument, if we followed COM-B model of behaviour change to design and implement interventions for this age group, how can the study results help us identify where capabilities, opportunities and motivations lie and how can adolescents be supported in their lacking space to change their behaviour. I think the rich discussion in this area makes this paper more useful.
   
  - **Response:** Some information on the applicability of the research findings exist in the discussion and is linked to the implications for Sustainable Development Goals and School Health Programmes by the Government of India. Further information has been added on the underlying theory of change models and linked with recommendations for the Government. Few comparative literatures has also been deleted.
2. A confusion/error, p.13, para 5: Seems to be a confusion between “price elasticity” and “income elasticity”. Please revise the sentence.
   
  - **Response:** As suggested, revised.
3. Limitations: Acknowledge that statistical tests are indicative only as the whole sample was purposive.
   
  - **Response: **Suggestion accepted and this has been incorporated in the limitation section.

**Conclusion**

1. Strongly recommend revising the conclusion section. The sentences here must be backed up by the study findings and leave any speculative policies to the Discussion section above. An example conclusion may be: “In the two states of India, a higher proportion of school students know the harm of tobacco use than the benefits of a healthy diet and physical activity. Both gender of the student and which type of the school that the student went to influenced specific KABs but both the magnitude and direction of such influence were variable. The data suggests that there is an urgent need for designing and implementing interventions that can improve school students’ capability and motivation as well as create opportunities to change their unhealthy behaviours.
   
  - **Response:** Suggestion accepted and the conclusion has been revised.
